# Prepandemic Antivaccination Websites' COVID-19 Vaccine Behavior: Content Analysis of Archived Websites

**DOI:** 10.2196/40291

**Published:** 2023-01-11

**Authors:** Samantha Kaplan, Megan von Isenburg, Lucy Waldrop

**Affiliations:** 1 Duke University Medical Center Library & Archives Durham, NC United States

**Keywords:** antivaccination behavior, web archiving, content analysis, COVID-19 vaccines, COVID-19, vaccine, website, web, pandemic, safety, science, content

## Abstract

**Background:**

The onset of the COVID-19 pandemic and the concurrent development of vaccines offered a rare and somewhat unprecedented opportunity to study antivaccination behavior as it formed over time via the use of archived versions of websites.

**Objective:**

This study aims to assess how existing antivaccination websites modified their content to address COVID-19 vaccines and pandemic restrictions.

**Methods:**

Using a preexisting collection of 25 antivaccination websites curated by the IvyPlus Web Collection Program prior to the pandemic and crawled every 6 months via Archive-It, we conducted a content analysis to see how these websites acknowledged or ignored COVID-19 vaccines and pandemic restrictions. Websites were assessed for financial behaviors such as having storefronts, mention of COVID-19 vaccines in general or by manufacturer name, references to personal freedom such as masking, safety concerns like side effects, and skepticism of science.

**Results:**

The majority of websites addressed COVID-19 vaccines in a negative fashion, with more websites making appeals to personal freedom or expressing skepticism of science than questioning safety. This can potentially be attributed to the lack of available safety data about the vaccines at the time of data collection. Many of the antivaccination websites we evaluated actively sought donations and had a membership option, evidencing these websites have financial motivations and actively build a community around these issues. The content analysis also offered the opportunity to test the viability of archived websites for use in scholarly research. The archived versions of the websites had significant shortcomings, particularly in search functionality, and required supplementation with the live websites. For web archiving to be a viable source of stand-alone content for research, the technology needs to make significant improvements in its capture abilities.

**Conclusions:**

In summary, we found antivaccination websites existing prior to the COVID-19 pandemic largely adapted their messaging to address COVID-19 vaccines with very few sites ignoring the pandemic altogether. This study also demonstrated the timely and significant need for more robust web archiving capabilities as web-based environments become more ephemeral and unstable.

## Introduction

### Background

The instability and rapidly evolving nature of internet content have led to fears of a “digital dark age” [1]. These concerns have been compounded by increasing misinformation, “fake news,” politicized science, and notable cases of social media companies acting without transparency to suppress content [2]. Web archiving tools offer an opportunity to capture and preserve some of this content before it evaporates. It is particularly vital to collect websites that illustrate varying viewpoints in the fiercest contemporary debates, as this content will continue to hold relevance to contemporary discourse.

With that in mind, *The Vaccination in Modern America: Misinformation vs. Public Health Advocacy Web Archive* was collaboratively developed by librarians from 2 health sciences libraries (Duke University and the University of Pennsylvania) whose institutions prioritize vaccine research and are members of the Ivy Plus Libraries Confederation [3]. The collection was launched publicly in February 2020 and currently includes 32 websites from pro- and antivaccination perspectives. Websites are still added to the collection and are selected based on relevance and the likelihood that they are not being preserved elsewhere. Weeks after the initial launch of the collection, the World Health Organization declared COVID-19 a global pandemic and much of the United States and the world went into lockdown. Unbeknownst to many, vaccine development had already begun [4].

The COVID-19 pandemic is unique in innumerable ways, but particularly in the vaccines created to combat it. While much has been made of the record time in which the vaccines were created [4], there are many other factors to consider. Worldwide interest and media coverage of the vaccines have been sustained and extensive. There are also multiple vaccines of varying efficacy developed by different pharmaceutical companies and scientists. Public knowledge of these options is significantly different from previous vaccines, which had limited choice or brand recognition. Subsequently, we must consider that this is a disease that has dominated the lived experience of the entire world for over 2 years. Historically, when cultural memory of a disease fades, so does vaccination willingness [5]. Unlike other illnesses, COVID-19 has also come with substantial misinformation and outright disbelief that the illness is real, even from individuals dying from the infection [6]. With these elements in mind, we aimed to see how previously identified antivaccination websites in the IvyPlus collection had or had not addressed the pandemic and COVID-19 vaccines via a content analysis. A secondary aim of this work is to assess the strengths and weaknesses of using archived websites in the context of research.

### Archiving the Fragile and Abundant Internet

The amount of freely accessible information on the World Wide Web is staggering, but that content is as fragile as it is ubiquitous and amorphous. The web, a focal point of culture in our society, is where we exchange ideas, share information, and document the day-to-day activities of life in the 21st century. In addition to the prominence the web plays in the production and support of culture, the distribution of its content happens swiftly with no geographic limitations. The only requirement to read or to contribute to the web is access to internet service or a cellular data plan.

While it is democratic in nature, the web is large with often undefined boundaries that must be viewed within context. A single website, removed from this interconnectedness, loses its context and value, unlike discrete digital files which have set parameters and established best practices for preservation [7]. In theory, a user types or selects a URL and is taken to that website, but, all too often, in practice, the published content at that web address has either changed or no longer exists. It is this rapidly changing landscape of the web that is at the nexus of the need to preserve its content. For example, after 1 year, 80% of webpages are no longer available as they were originally published [8], and, after 27 months, 13% of web references in scholarly articles disappear [9]. Without the resources to capture and save this web content, gaps in the historical record are inevitable.

This is problematic as web content has enduring historic value and should be saved, preserved, and made accessible for future generations. In 2003, UNESCO’s “Charter on the Preservation of Digital Heritage” recognized the importance of its preservation, stating “that the disappearance of heritage in whatever form constitutes an impoverishment of the heritage of all nations” [10]. The importance of capturing and preserving these resources goes beyond saving the content only for historical value and research, as preserving web content also supports current technology by “assessing the trustworthiness of statements, detecting web spam, improving web information retrieval, [and] forecasting events” [11]. Without appropriate systems, this web content will be lost and the outcome will be not only a gap in our understanding of the past but also an inability to synthesize our present.

### The Role of Internet Archives and Archive-It

The Internet Archive is a nonprofit, founded in 1996, with the mission to create a digital archive to permanently store as much of the internet as possible [12]. This is done in 2 ways: Wayback Machine (free) and Archive-It (paid subscription) [13]. Archive-It, the subscription web archiving service for users, enables subscribing institutions, such as libraries, to curate collections of web content specific to their institution and collection development policies [13]. Archive-It services over 800 organizations in more than 24 countries. These organizations include “libraries, cultural memory and research institutions, social impact and community groups, and educational and open knowledge initiatives” [14]. To date, the amount of web content captured and preserved by users totals over “40 billion born born-digital, web-published records, totaling petabytes of data” [14]. Archive-It markets itself as easy to use and provides a full set of tools to capture web-based content, training on how to capture web-based content, as well as technical support for any issues encountered by their subscribers.

### The Role of IvyPlus Digital Archives

Among the over 800 organizations that Archive-It services is the Ivy Plus Libraries Confederation (IPLC), a group of 13 academic libraries with the mission to benefit current and future scholars globally by leveraging their “collective assets to ….. shape the discourse around scholarly communication, and the outcomes of that discourse” [3]. Established in 2017, the IPLC Web Collecting Program “is a collaborative collection development effort to build curated, thematic collections of freely available, but at-risk, web content in order to support research at participating Libraries and beyond” [3].

The IPLC maintains 30 public collaborative and thematic collections documenting at-risk web content across the globe. In order to be considered for inclusion, the selected websites must be freely available (no logins or paywalls), must exclude institutional content belonging to confederation institutions, and all partner libraries must maintain at least 1 Archive-It account at the institutional level. Once the collections are established and the websites are added, the bulk of the IPLC web archiving collections is crawled twice a year [3].

In February 2020, the IPLC Web Collecting Program launched *The Vaccination in Modern America: Misinformation vs. Public Health Advocacy Web Archive.* The Archive's scope is as follows [15]:

The Archive identifies and captures webpages representing the current state of public discourse and contrasting approaches to authority on the topic in the United States, with a focus on sites that are both pro- and anti-vaccination. The purpose of the collection is to capture potentially ephemeral information about vaccination that could be used by health service researchers, information scientists, sociologists, and others to understand the motivations, practices, and outcomes of health information and misinformation. Anti-vaccination sites were those that opposed the established national guidelines for vaccination and vaccination schedules, as well as those that focus on chemical vaccine adjuvants or organic natural living as a replacement for vaccination. Prominent natural living advocates and bloggers who have made anti-vaccination arguments, or who have expressed sympathies to vaccine avoidance, have been included to provide additional context around anti-vaccination information campaigns.

This web archive includes both pro- and antivaccination websites that are crawled every 6 months by Archive-It’s web crawling technology, saved, and made publicly available. Researchers in public health and computer science were consulted for recommendations on antivaccination websites for inclusion. Medical librarians, including the 3 authors, selected the websites to be crawled, wrote the descriptive metadata, and assigned appropriate subject headings to each website in order to assist with discovery by future researchers. The project was approached from public health, misinformation, and archival perspectives and opposes the rhetoric of the antivaccination websites. In the ensuing years, this collaborative web collection will continue to grow as more crawls are completed and saved, facilitating access to transient websites that will undoubtedly change or disappear.

### Antivaccination Behavior on the Internet

Content analyses of antivaccination content are not uncommon, with studies using data from multiple sources, including search results [16], Facebook groups [17], specific websites [18], and Pinterest [19], to name a few. Uniformly, these analyses find that antivaccination content has a larger digital presence than provaccination content.

Many researchers have worked to distill web-based antivaccination behavior into common themes, discrete categories, or behavioral techniques. Kata [20] identifies 4 tactics used by the antivaccination movement on the internet: skewing the science, shifting hypotheses, censorship, and attacking the opposition. These tactics all work in tandem to appeal to “the most commonly cited reason for general population hesitancy towards vaccination … safety concerns” [21]. The reasons for fearing the safety of vaccines are bountiful and have been well enumerated by other researchers [21]. Some of these fears are not well-founded and perpetuate harmful and ableist claims, such as the much discredited link between the measles, mumps, and rubella vaccine and autism spectrum disorder [22]. Antivaccination activists also appeal to vaccine-hesitant individuals by advocating for personal autonomy over one's body [23], an argument that has found new relevance during COVID-19 debates about mask and vaccine mandates. Another common trope of antivaccination content is distrust of institutions [21], ranging from regulatory bodies, health care organizations, corporations, and others.

It is important to examine how antivaccine websites are responding to the COVID-19 pandemic, which is different from other vaccine-preventable illnesses. Typically, when researching antivaccine behavior, we must acknowledge, “fewer persons have ever witnessed a child who is severely ill with a vaccine-preventable disease” [24], whereas currently, there have been over 80 million American COVID-19 infections and over 1 million have died [25]. In another departure from typical vaccine behavior, researchers usually study parental attitudes toward vaccines for their children, but COVID-19 has created a situation where teenagers are pursuing avenues to receive COVID-19 vaccines without parental consent [26]. There has also been unique politicization of COVID-19, compared to other vaccine-preventable diseases [27].

### Objective

This study had 2 primary objectives. The first aim was to document how antivaccination websites that predated the COVID-19 pandemic addressed the pandemic in their content. The second objective was to test the viability of using archived versions of websites as sources of data for a content analysis, as opposed to the live versions.

## Methods

### Content Selection

To determine how antivaccination websites have addressed the COVID-19 vaccine, we conducted a content analysis of the antivaccination websites in *The Vaccination in Modern America Web Archive* (n=25). This curated archive of sites is not simply a convenience sample: each site was previously vetted for inclusion in the *Vaccination in Modern America Web Archive* by both the authors and additional information specialists working with the IvyPlus Web Archive program. The collection includes archived versions of all indexed sites representing content from 2019 to the present, offering a rare longitudinal presentation of antivaccination content from before and during the COVID-19 pandemic. This archive is freely available to other researchers by visiting the Ivy Plus Libraries Confederation website and filtering by the metadata tag for “Anti-vaccination.” Within the archive, this is a binary filter, all included websites are tagged as ”Anti-vaccination“ or “Pro-vaccination.” The full list of websites included is available in Multimedia Appendix 1.

The coding schema was informed by common antivaccination themes identified by Kata [20], safety concerns identified by [28], and the authors. The coding schema and codebook are available in Multimedia Appendix 2. Websites were assessed across broad categories, such as COVID-19 vaccines (whether they mentioned them at all or by brand name), personal freedom (masking, bodily integrity, stay-at-home orders, etc), safety concerns (compromised research process, inclusion of specific ingredients, etc), and distrust of science and institutions (financial motivations, corruptions, biological warfare, etc). If an attribute was present on a website, the authors coded it with a “Y,” if not an “N,” and a “U” if unclear.

The authors utilized a Google Sheet to collect the data. All 3 authors participated in a pilot round to test the coding schema in which they evaluated the same 3 websites and compared their findings. This pilot resulted in minor structural adjustments to the codebook, primarily to clarify the definition of certain attributes.

All websites were viewed and coded by 2 researchers, with each author reviewing 16 or 17 websites (approximately two-thirds). Coding was conducted during a 3-week period (December 20, 2021, to January 7, 2022) agreed upon by all authors. This was important because authors would potentially be using live websites to supplement the archived versions and needed to be assessing the live versions during the same time period. Authors primarily used the archived versions of the websites to test the ability of the web archive to support future research but supplemented this with live versions of the sites. Authors would first go to the archived versions to assess them for all attributes of the codebook. Often, the archived versions had such limited search function or just the homepage had been well preserved, it was necessary to then go to the live version of the website to look for other attributes that might be addressed in website subpages. Because the unit of analysis was the website, rather than the individual webpage, we wanted to be exhaustive and comprehensive when looking to see if a website had the presence of an attribute. If an attribute was observed on an archived version of the website, but not the present day version, it was still counted. Interrater agreement was 80%, and mediation was conducted by the first author who also performed descriptive statistics. Conflicts were resolved via first author if presence was easily established, group consensus was sought if further examination was warranted (eg, clarifying criteria, particularly around presence at all vs negative presence).

### Ethical Considerations

The authors did not seek IRB approval as this was not human subjects research. All of the websites observed and their archived versions were publicly available.

## Results

### Summary of Results

**Figure 1:**
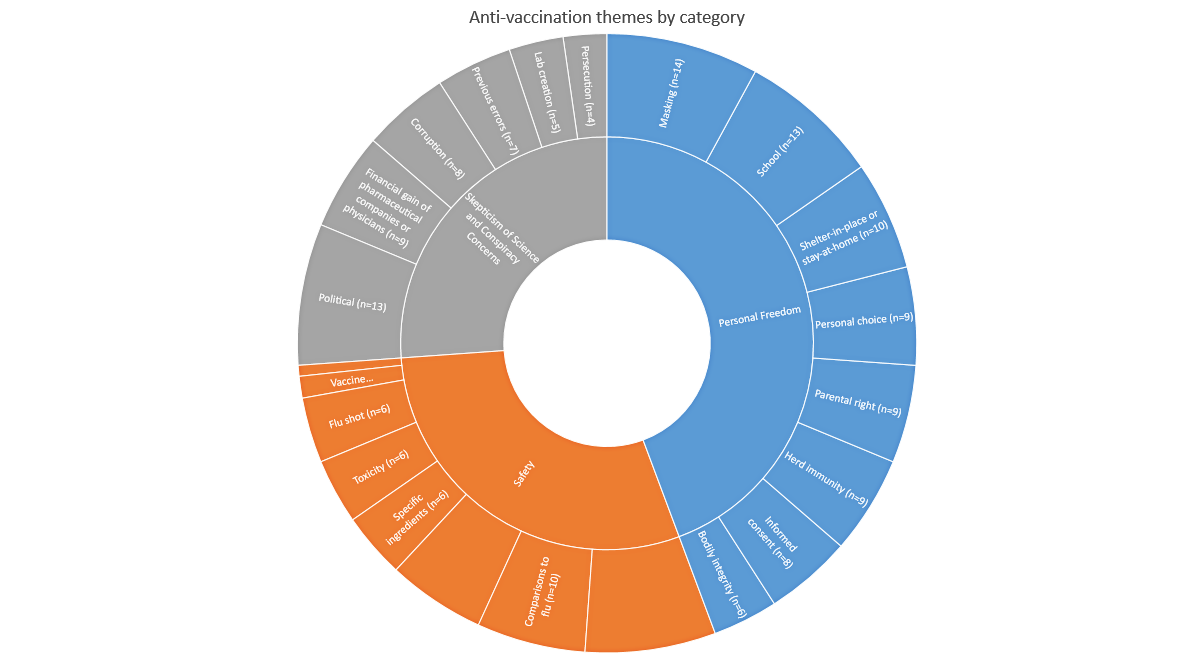
Summary of results.

All results are summarized in Figure 1, raw data for this figure are available in Multimedia Appendix 3.

### Financial Gain

More than half (14, 56%) of sites solicited donations and about half (n=12, 48%) mention their tax status. We did not collect tax status details, but sites largely purported to be nonprofits. In addition to collecting donations, 18 (72%) sites had a membership option of some kind, be it allowing individuals to pay to join or sign up for a free or paid newsletter. Web-based newsletters have recently made headlines for their lucrative potential, with 1 COVID-19 misinformation newsletter netting upward of US $30,000 a month [29]. About a quarter (n=6, 24%) of sites were also set up to draw income from Amazon, as members of the Amazon affiliates program or beneficiaries of AmazonSmile (a program that allows shoppers to donate a portion of their purchase to the charity of their choice).

### COVID-19 Vaccines

About two-thirds (n=16, 64%) of sites address COVID-19 vaccines in some way and more than one-third mentions at least 1 COVID-19 vaccine with Moderna being the most commonly mentioned (n=11, 44%), closely followed by Pfizer (n=9, 36%). Only 2 (8%) sites mentioned vaccines other than Pfizer, Moderna, Johnson & Johnson, or AstraZeneca.

### Antivaccination Themes

Overall, more than half of the sites in *The Vaccination in Modern American Web Archive* referenced antivaccination themes related to COVID-19, with 16 (64%) sites referencing some element of personal freedom, 15 (60%) referencing skepticism of science and conspiracy concerns, and 52% referencing safety; 3 (12%) did not mention any antivaccination themes, but all 3 (12%) of these sites were static and had not been updated since before the pandemic began or no longer went to working websites.

### Infringement on Personal Freedom

More than half of sites mentioned at least one personal freedom attribute with masking being the most frequent (n=14, 56%) and bodily integrity the least (n=6, 24%).

### Skepticism of Science

More than half of sites expressed skepticism of politicians’ motivations for various COVID-19 and COVID-19 vaccine policies. About one-third (n=9, 36%) of sites alluded to the financial motivations of pharmaceutical companies or physicians as driving COVID-19 vaccine development or uptake, and a similar amount mentioned corruption (n=8, 32%). Relatively fewer websites (n=4, 16%) mentioned persecution or fear of persecution for COVID-19 vaccine hesitancy or refusal. Previous errors, such as the Cutter Incident [29], were mentioned by 7 (28%) sites. The idea that COVID-19 emerged from a research laboratory was mentioned by only 5 sites.

### Safety of COVID-19 Vaccines

Very few sites mentioned COVID-19 vaccination in the context of the pediatric immunization schedule (n=2, 8%) or concerns about a causal relationship between COVID-19 vaccines and autism spectrum disorder (n=1, 4%). This could potentially be attributed to our data collection period occurring before pediatric vaccines were widely available. Given the early pandemic comparisons between COVID-19 and seasonal influenza, the high percentage of sites making comparisons to the flu (n=10, 40%) was not surprising. About half (n=12, 48%) of sites mentioned adverse effects from or specific ingredients of concern (n=6, 24%) regarding COVID-19 vaccines; it was outside of the scope of this study to collect the unique adverse effects or ingredients mentioned. In aggregate, 13 (52%) of sites mentioned at least 1 safety concern, but no singular safety concern was mentioned by these many sites. This suggests that at the point of data collection, COVID-19 vaccine safety concerns had not yet coalesced around a specific concern in the way that certain pediatric vaccines have.

## Discussion

### Antivaccine Website Practices

The financial aspect of antivaccine web behavior must be addressed. Nearly half of the sites mentioned their tax status (almost universally some type of nonprofit) and solicited donations, with these attributes occurring together in 12 (48%) sites. While this is common for nonprofits, few of these sites had additional markers of authenticity (such as a Charity Navigator rating) or information regarding how they spend their funds. It is unclear if the sites’ portrayals of themselves as persecuted outsiders are a financial necessity or financially canny, as 7 (28%) sites that requested donations also questioned the financial motives of pharmaceutical companies and physicians. Based on our observations, websites are using traditional symbols of trustworthiness to gain income but are subject to minimal or no oversight. While outside the scope of content analysis, future research could analyze site profitability and spending.

At least one-fourth of our sites were affiliated with Amazon in some way. This is not the first time Amazon’s practices have allowed problematic groups or sellers to profit [30], and it adds further evidence that large corporations are perhaps not discriminatory when allowing websites to participate in programs. For example, although not part of our content analysis, we did observe Google ads on many sites. Many advertisers are not always aware of what types of sites their ads may be shown on, as observed by activists advocating directly to brands to blacklist Breitbart in 2016 [31].

Sites represented nonprofits, political advocacy groups, and individuals. Individuals’ sites generally fell into 2 categories—blogs that operationalized a parental identity or sites that traded on the individual’s traditional professional expertise or background. The latter are particularly interesting as they utilize “insider” characteristics, such as an MD degree or experience working with health care, to legitimize “outsider” ideas, such as collusion and corruption by pharmaceutical companies and the government. There is an irony in someone with the title and credentials of a physician using those very means to impugn a profession without disavowing their own membership and aligns with Kata's finding that ”expert knowledge may be treated as part of the problem“ [23]. These findings provide a ”more nuanced understanding of the interplay between vaccination attitudes, social network structure, and information sources, including actors with a vested interest in promoting false beliefs“ [32] called for by a large study of antivaccination discourse on Twitter. Of further concern, doing so appears to be very profitable and exploitative of vulnerable people. Health care personnel and organizations should consider becoming familiar with more predominant personalities in this area to be more prepared to confront the issue.

Individual blogs appeared more likely to “die” or cease creating new content, whereas the more professional sites had a tendency to “molt” and emerge as something new or be subsumed by larger sites. The absorption into or redirect to existing sites underscores previous findings that disinformation sites amplify their voices by spreading their messaging across multiple sites [33]. This underscores that the internet is an interconnected ecosystem in which problematic content can exploit its durability. Similar to a hydra, snuffing out one website or head only creates an opportunity for 2 new ones to emerge.

### COVID-19 Vaccine Hesitancy

Representations of COVID-19 vaccine hesitancy were heterogeneous. Even with the unique option of being able to select a vaccine option from multiple pharmaceutical companies, trust in vaccines did not appear to increase. Specific vaccines were not used to rank 1 option as more preferable to another but were rather used to discredit the notion in general. This aligns with prior studies that have observed that vaccine hesitancy is more about vaccine type, number of vaccines, or vaccine ingredients [34] than the manufacturer. This is interesting given the number of sites that mentioned concerns about financial motivations on the part of pharmaceutical companies, a trope identified as “You're in the pocket of Big Pharma” by Kata [20]. While this study did not quantify mentions of vaccines beyond Pfizer, Moderna, AstraZeneca, and Johnson & Johnson, very few other vaccine manufacturers were observed. This could be attributed to this study’s sample being restricted to English-language sites.

Some of the sites included in our sample were single issues, such as the Immunity Resource Foundation which focuses on HIV/AIDS and did not create unique COVID-19 content or absorb aspects of COVID-19 into their messaging. Other sites expressed specific but variable vaccine concerns in regard to COVID-19, but not all attributes, underscoring the heterogeneity of vaccine hesitancy does extend to COVID-19 vaccine hesitancy. Messaging over school policies was negative but for conflicting reasons. Some sites' content resisted school closings, others resisted reopenings, and there was consistently anticipatory fear that children would be required to receive the COVID-19 vaccine to attend. This provides evidence for specialized outreach around COVID-19 vaccines to increase uptake and address concerns. This aligns with the findings of a scoping review of 50 studies which asserted vaccination attitudes toward COVID-19 vaccines are “shaped by factors that are multifaceted and multilevel” [35]. We did not collect more granular data in this area, but future studies should consider exploring this, as there is significant tension between the right to receive a public education, the delivery of that education, and the belief to determine the health care choices for one's child.

### Web Archiving: Promises and Pitfalls

Kata's 2010 content analysis of antivaccine search results noted ”This analysis was limited by the transient nature of the Internet, where websites addresses and search rankings constantly change“ [23]. In theory, web archiving could provide a balm to this issue; in practice, using a web archive for this content analysis presented some challenges. Based on our experiences using the Archive-It captures, web archiving misses or inconsistently captures numerous important elements of a website, particularly search, graphics, social media (the Ivy Plus Web Collecting Program does not crawl social media regardless of Archive-It's ability to capture it), video, pay-walled content, capture of external links, and content timed to disappear. Some content simply cannot be crawled without manual assistance. Since search functionality is limited in the archived sites, determining the presence of an element remains challenging. Most of our sites had a membership option of some kind, be it an email newsletter or access to subscriber-only content. This subscription-based content is not being captured by web archiving and is thus being lost as content creators move content from websites to paid newsletter models, podcasts, or social media. This results in only the most permanent and perhaps palatable content being preserved by web archiving tools with rich information being lost to time.

**Figure 2:**
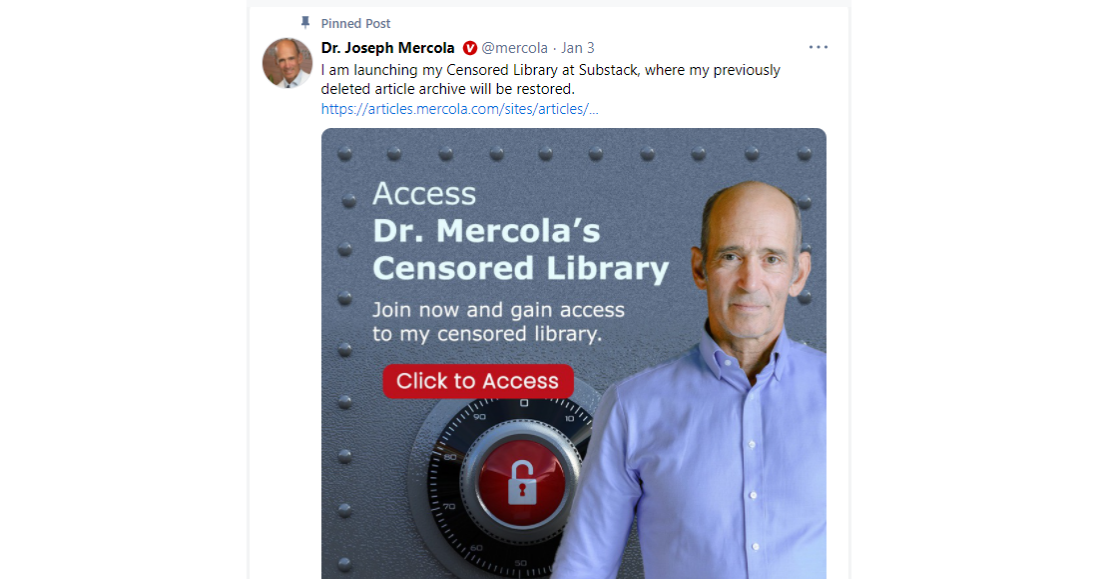
Dr Mercola's censored library.

During our data collection, it became clear that creators of problematic content are adapting to “receipts” culture, particularly with premium models to monetize content and make it harder to access. Showing “receipts” comes from Black culture and is a method to demand or demonstrate accountability based on documentation [36]. This means certain websites' stability has made them threatening. For example, old and racist Tweets have led to so many untimely resignations that scrubbing one's web-based presence has become an entire industry. Rather than consider the long-term implications of their words, creators are building obsolescence into their content. For evidence of this, see Figure 2, from Dr Mercola's Twitter post about their website. Active erasure of problematic content is being built in. The ability of creators to curate and erase or revise their images over time has rapidly outpaced the tools we have to capture records for posterity, making it difficult if not impossible to trace.

This highlights the limits of web archiving and how much ephemeral web content is potentially lost. Our content analysis was frequently obstructed by limitations with Archive-It, such as limited capture of externally hosted content or subscriber-only content. This is concerning as Archive-It is the primary tool relied upon for the collection and curation of websites for future research by scholars [37]. There are very few other options available, one example is Conifer, emphasizing the need for further development. Some of this may be attributed to the limited number of user experience studies on Archive-It, underlining the need for more research into the development and use of web archives for scholarship [37].

While we have focused our attention on Archive-It, there are other avenues to consider. We know there is extensive web-based scholarly research occurring constantly across sites, networks, and platforms, for example, researchers built a public data set of antivaccine Twitter content [38]. Perhaps, it is time to consider a repository of web data for archival purposes, including research data reuse. No matter what, there needs to be more web archiving—more frequent, more comprehensive, and more voluminous is urgently needed. Web archives need to be deeper, richer, and more accessible. Records write history and we risk losing precious context for important events. To achieve this, web archiving tools need to be enhanced to capture the interconnected information, including social media, subscription or pay-walled content, search, and videos on sites.

### Limitations

This study has several significant limitations. Our sample was derived from an archived collection of websites curated by medical librarians and archivists, whose scope was to capture a wide range of opinions, there are undoubtedly more and public health personnel might have considered other viewpoints valuable. Our pool of websites was also limited to sites that had existed prior to the emergence of the COVID-19 pandemic and the vaccines and had been archived. Our data are not reflective of websites, which have emerged specifically due to COVID-19 vaccine hesitancy. We did not quantify site type because our sample was predetermined by the collection curators (of which the authors are members) and cannot be representative of the actual landscape of these types of sites. Our research was also hindered by the content we were unable to access because it was not captured by the web archiving tools or due to paywalls limiting access. Our data also do not include any social media websites, which are established to be rife with vaccine misinformation [39]. Given the amount of content we could not access, the results here are likely an underrepresentation.

This study's findings are also limited by the narrow nature of the coding schema, which aimed to determine if the presence of established antivaccination tropes redirected toward COVID-19. We did not capture or create COVID-19–specific elements or conduct any open or inductive coding to generate COVID-19–specific attributes for our content analysis. Therefore, this study does not contribute a unique analysis of COVID-19 vaccine hesitancy. Future studies should attempt to capture information on vaccine boosters, female infertility concerns, antivaccine sites’ relationship with fringe social networks like Gab, and the citing of authoritative sources, such as the Centers for Disease Control. This also highlights some of the challenges of researching an active phenomenon, as once data collection had begun, we did not adjust our codebook to allow for significant changes to COVID-19 policies (such as a wide-scale implementation of COVID-19 boosters in the United States or changes to quarantine protocols). Certain common tropes in antivaccination activism, such as persecution and prior scientific errors [20], were not evident in this study; however, they may emerge over time as COVID-19 vaccine hesitancy is still nascent and will continue to evolve.

### Conclusions

Librarians and information professionals need to create more curated and accessible archives of ephemeral web content. Web archives can provide detailed data, particularly over time, which illustrate the rich tapestry of everyday life. To achieve this, web archiving tools must improve in precision, comprehensiveness, and search and retrieval. *The Vaccination in Modern America Web Archive* creates the opportunity for longitudinal study of COVID-19 vaccine hesitancy. For example, we noticed about half (48%) of sites mentioned adverse effects and only 8% mentioned the pediatric immunization schedule. This makes sense given that at the time of data collection, most available information about COVID-19 vaccines related to outcomes from the randomized controlled trials to test the vaccine efficacy, and there was little to no conversation about incorporating the vaccine into the schedule for children. Future studies could examine how these sites' messaging changes and potentially create a timeline to model the development of opposition to a specific vaccine.

In-depth study of COVID vaccine hesitancy is needed, although our findings do demonstrate this behavior aligns somewhat with established vaccine hesitancy information behavior.

## Appendix

Multimedia Appendix 1 Included Websites.

Multimedia Appendix 2 Codebook.

Multimedia Appendix 3 Raw data for Figure 1.

## Abbreviations

IPLC: Ivy Plus Libraries Confederation

## References

[1] Jeffrey S (2012). A new digital dark age? Collaborative web tools, social media and long-term preservation. World Archaeol.

[2] Angwin J, Grassegger H, ProPublica (2017). Machine bias: Facebook’s secret censorship rules protect white men from hate speech but not black children. ProPublica.

[3] (2022). Ivy Plus Libraries Web Collecting Program. Ivy Plus Libraries Confederation.

[4] Ball P (2020). The lightning-fast quest for COVID vaccines—and what it means for other diseases. Nature.

[5] Baumgaertner B, Ridenhour BJ, Justwan F, Carlisle JE, Miller CR (2020). Risk of disease and willingness to vaccinate in the United States: a population-based survey. PLoS Med.

[6] Shannon J (2020). 'It's not real': in South Dakota, which has shunned masks and other COVID rules, some people die in denial, nurse says. USA Today.

[7] Post C (2017). Building a living, breathing archive: a review of appraisal theories and approaches for web archives. Preserv Digit Technol Cult.

[8] Ntoulas A, Cho J, Olston C (2004). What's new on the web?: the evolution of the web from a search engine perspective.

[9] Dellavalle R, Hester EJ, Heilig LF, Drake AL, Kuntzman JW, Graber M, Schilling LM (2003). Going, going, gone: lost internet references. Science.

[10] UNESCO (2003). Records of the General Conference, 32nd session, Paris, 29 September to 17 October.

[11] Costa M, Gomes D, Silva MJ (2017). The evolution of web archiving. Int J Digit Libr.

[12] (2022). About the Internet Archive. Internet Archive.

[13] (2022). Wayback Machine. Internet Archive.

[14] (2014). About the internet archive. Archive-It.

[15] Ivy Plus Libraries Confederation (2020). Vaccination in modern America: misinformation vs. public health advocacy. Archive-It.

[16] Wolfe RM, Sharp LK (2005). Vaccination or immunization? The impact of search terms on the internet. J Health Commun.

[17] Buchanan R, Beckett RD (2014). Assessment of vaccination-related information for consumers available on facebook. Health Info Libr J.

[18] Davies P, Chapman S, Leask J (2002). Antivaccination activists on the world wide web. Arch Dis Child.

[19] Guidry JP, Carlyle K, Messner M, Jin Y (2015). On pins and needles: how vaccines are portrayed on pinterest. Vaccine.

[20] Kata A (2012). Anti-vaccine activists, web 2.0, and the postmodern paradigm: an overview of tactics and tropes used online by the anti-vaccination movement. Vaccine.

[21] Yaqub O, Castle-Clarke S, Sevdalis N, Chataway J (2014). Attitudes to vaccination: a critical review. Soc Sci Med.

[22] Offit PA, Coffin SE (2003). Communicating science to the public: MMR vaccine and autism. Vaccine.

[23] Kata A (2010). A postmodern Pandora's box: anti-vaccination misinformation on the internet. Vaccine.

[24] Feikin DR, Lezotte DC, Hamman RF, Salmon DA, Chen RT, Hoffman RE (2000). Individual and community risks of measles and pertussis associated with personal exemptions to immunization. JAMA.

[25] (2022). COVID Data Tracker. US Department of Health and Human Services, CDC.

[26] Morgan L, Schwartz JL, Sisti DA (2021). COVID-19 vaccination of minors without parental consent: respecting emerging autonomy and advancing public health. JAMA Pediatr.

[27] Chen HF, Karim SA (2021). Relationship between political partisanship and COVID-19 deaths: future implications for public health. J Public Health (Oxf).

[28] Alba D (2022). The latest covid misinformation star says he invented the vaccines. The New York Time.

[29] Fitzpatrick M (2017). The cutter incident: how America's first polio vaccine led to a growing vaccine crisis. J R Soc Med.

[30] Doubek J (2018). Amazon pulls some Nazi-themed, offensive items after criticism, but many remain. NPR.

[31] Johnson E (2018). How a Twitter account convinced 4,000 companies to stop advertising on Breitbart. Vox.

[32] Mønsted B, Lehmann S (2022). Characterizing polarization in online vaccine discourse: a large-scale study. PLoS ONE.

[33] Mabrey BE (2021). The disinformation dozen and media misinformation on science and vaccinations(thesis). Oregon State University.

[34] Nadeau JA, Bednarczyk RA, Masawi MR, Meldrum MD, Santilli L, Zansky SM, Blog DS, Birkhead GS, McNutt LA (2015). Vaccinating my way: use of alternative vaccination schedules in New York State. J Pediatr.

[35] Al-Jayyousi GF, Sherbash MAM, Ali LAM, El-Heneidy A, Alhussaini NWZ, Elhassan MEA, Nazzal MA (2021). Factors influencing public attitudes towards COVID-19 vaccination: a scoping review informed by the Socio-Ecological Model. Vaccines (Basel).

[36] Brekke AJ, Joseph R, Aaftaab NG (2021). “I address race because race addresses me”: women of color show receipts through digital storytelling. Rev Commun.

[37] Abrams S, Antracoli A, Appel R, Caust-Ellenbogen C, Denison S, Duncan S, Ramsay S (2019). Sowing the seeds for more usable web archives: a usability study of Archive-It. Am Archivist.

[38] Muric G, Wu Y, Ferrara E (2021). COVID-19 vaccine hesitancy on social media: building a public twitter data set of antivaccine content, vaccine misinformation, and conspiracies. JMIR Public Health Surveill.

[39] Burki T (2020). The online anti-vaccine movement in the age of COVID-19. Lancet Digit Health.

