# Supplementary material for: Prepandemic Antivaccination Websites' COVID-19 Vaccine Behavior: Content Analysis of Archived Websites
Source: JMIR Form Res. 2023 Jan 11;7:e40291. doi: 10.2196/40291 (PMC9838720; doi:10.2196/40291)
Supplement: Multimedia Appendix 1 [file formative_v7i1e40291_app1.docx]

Multimedia Appendix 1: Included Websites

| Health Freedom Action | [http://healthfreedomaction.org/](https://wayback.archive-it.org/13389/*/http:/healthfreedomaction.org/) |
| --- | --- |
| Vaccine Awareness Network | [http://vaccineriskawareness.com/](https://wayback.archive-it.org/13389/*/http:/vaccineriskawareness.com/) |
| Health Liberty | [http://www.health-liberty.org/](https://wayback.archive-it.org/13389/*/http:/www.health-liberty.org/) |
| Immunity Resource Foundation | [http://www.immunity.org.uk/](https://wayback.archive-it.org/13389/*/http:/www.immunity.org.uk/) |
| Vaccination Liberation | [http://www.vaclib.org/](https://wayback.archive-it.org/13389/*/http:/www.vaclib.org/) |
| Children's Health Defense | [https://childrenshealthdefense.org/](https://wayback.archive-it.org/13389/*/https:/childrenshealthdefense.org/) |
| Fearless Parent | [https://fearlessparent.org/](https://wayback.archive-it.org/13389/*/https:/fearlessparent.org/) |
| Immunity Education Group | [https://immunityeducationgroup.org/](https://wayback.archive-it.org/13389/*/https:/immunityeducationgroup.org/) |
| J.B. Handley Blog | [https://jbhandleyblog.com/](https://wayback.archive-it.org/13389/*/https:/jbhandleyblog.com/) |
| Modern Alternative Mama | [https://modernalternativemama.com/](https://wayback.archive-it.org/13389/*/https:/modernalternativemama.com/) |
| Ohio Advocates for Medical Freedom | [https://ohioamf.org/](https://wayback.archive-it.org/13389/*/https:/ohioamf.org/) |
| VacTruth | [https://vactruth.com/](https://wayback.archive-it.org/13389/*/https:/vactruth.com/) |
| Vaxxter | [https://vaxxter.com](https://wayback.archive-it.org/13389/*/https:/vaxxter.com) |
| Age of Autism | [https://www.ageofautism.com/](https://wayback.archive-it.org/13389/*/https:/www.ageofautism.com/) |
| Ask Dr. Sears | [https://www.askdrsears.com/](https://wayback.archive-it.org/13389/*/https:/www.askdrsears.com/) |
| Children's Medical Safety Research Institute | [https://www.cmsri.org/](https://wayback.archive-it.org/13389/*/https:/www.cmsri.org/) |
| Focus for Health | [https://www.focusforhealth.org/](https://wayback.archive-it.org/13389/*/https:/www.focusforhealth.org/) |
| Generation Rescue | [https://www.generationrescue.org/](https://wayback.archive-it.org/13389/*/https:/www.generationrescue.org/) |
| Learn the Risk | [https://www.learntherisk.org/](https://wayback.archive-it.org/13389/*/https:/www.learntherisk.org/) |
| Living Whole | [https://www.livingwhole.org/](https://wayback.archive-it.org/13389/*/https:/www.livingwhole.org/) |
| Michigan for Vaccine Choice | [https://www.michiganvaccinechoice.org/](https://wayback.archive-it.org/13389/*/https:/www.michiganvaccinechoice.org/) |
| Moms Across America | [https://www.momsacrossamerica.com/](https://wayback.archive-it.org/13389/*/https:/www.momsacrossamerica.com/) |
| National Vaccine Information Center | [https://www.nvic.org/](https://wayback.archive-it.org/13389/*/https:/www.nvic.org/) |
| Healthy Home Economist | [https://www.thehealthyhomeeconomist.com/](https://wayback.archive-it.org/13389/*/https:/www.thehealthyhomeeconomist.com/) |
| Weston A. Price Foundation | [https://www.westonaprice.org/](https://wayback.archive-it.org/13389/*/https:/www.westonaprice.org/) |
